# An Unusual Location of Osteochondroma: Dorsal Scapula

**DOI:** 10.7759/cureus.6464

**Published:** 2019-12-25

**Authors:** Yunus E Bektas, Ramadan Ozmanevra

**Affiliations:** 1 Orthopaedics and Traumatology, Kilis State Hospital Orthopaedics and Traumatology, Kilis, TUR; 2 Orthopaedics and Traumatology, University of Kyrenia, Dr. Suat Gunsel Hospital, Kyrenia, CYP

**Keywords:** scapula, osteochondroma, dorsal, unusual location

## Abstract

Osteochondromas commonly affect the proximal humerus, pelvis, and knee but are rarely seen on flat bones. Herein, we present the case of a 15-year-old female patient with osteochondroma located at the dorsal aspect of the scapula. The patient was admitted to the Orthopedics and Traumatology Department with the complaint of a mass on the left upper back for five years. The patient complained of the inability to sleep in the supine position, pain with shoulder motion, and cosmetic discomfort for two years. X-rays of the left shoulder revealed a bony mass arising from the dorsal aspect of the left scapula. The patient underwent an operation, and a specimen was sent for histopathologic examination. The histopathologic investigation confirmed the diagnosis of non-malignant transformation osteochondroma. While osteochondroma is not common in the scapula, it should be kept in mind that the most common benign tumor of the scapula is osteochondroma.

## Introduction

Osteochondroma is the most common type of benign bone tumor arising from the surface of a bone. Osteochondromas commonly affect the proximal humerus, pelvis, and knee but are rarely seen on flat bones [1]. However, the most common benign tumor of the scapula is osteochondroma [2-4].

Many cases of osteochondroma with ventral location causing pseudo-winging and snapping scapula have been reported in the literature [5-8]. In contrast to ventral location, there have been few reported cases of osteochondroma affecting the dorsal aspect of the scapula [9,10].

Herein, we present a case of osteochondroma located at the dorsal aspect of the scapula.

## Case presentation

**Figure 1:**
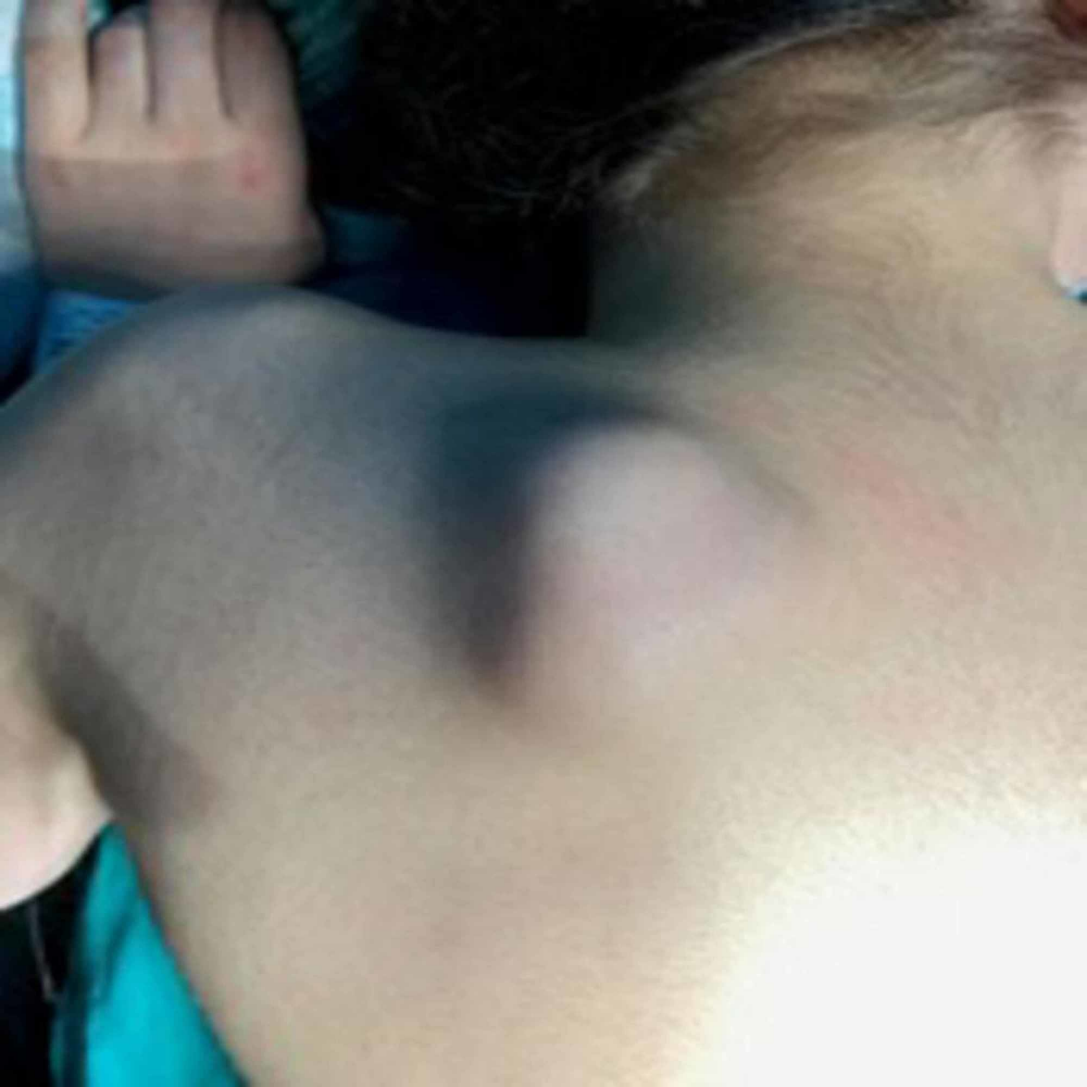
A hard, oval solid palpable mass on the superomedial of dorsal scapula.

**Figure 2:**
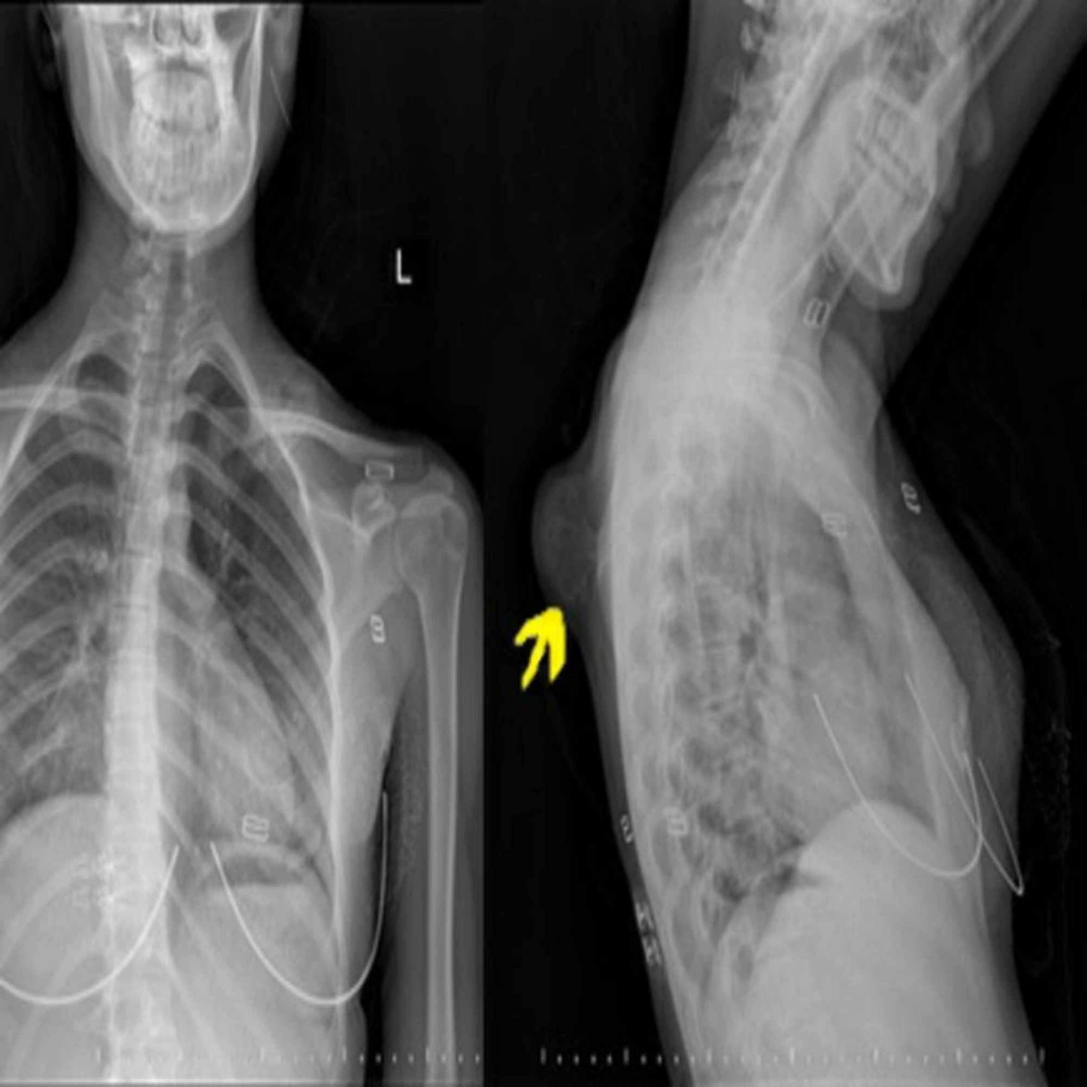
X-rays of the left shoulder revelead a bony mass arising from the dorsal aspect of the left scapula.

**Figure 3:**
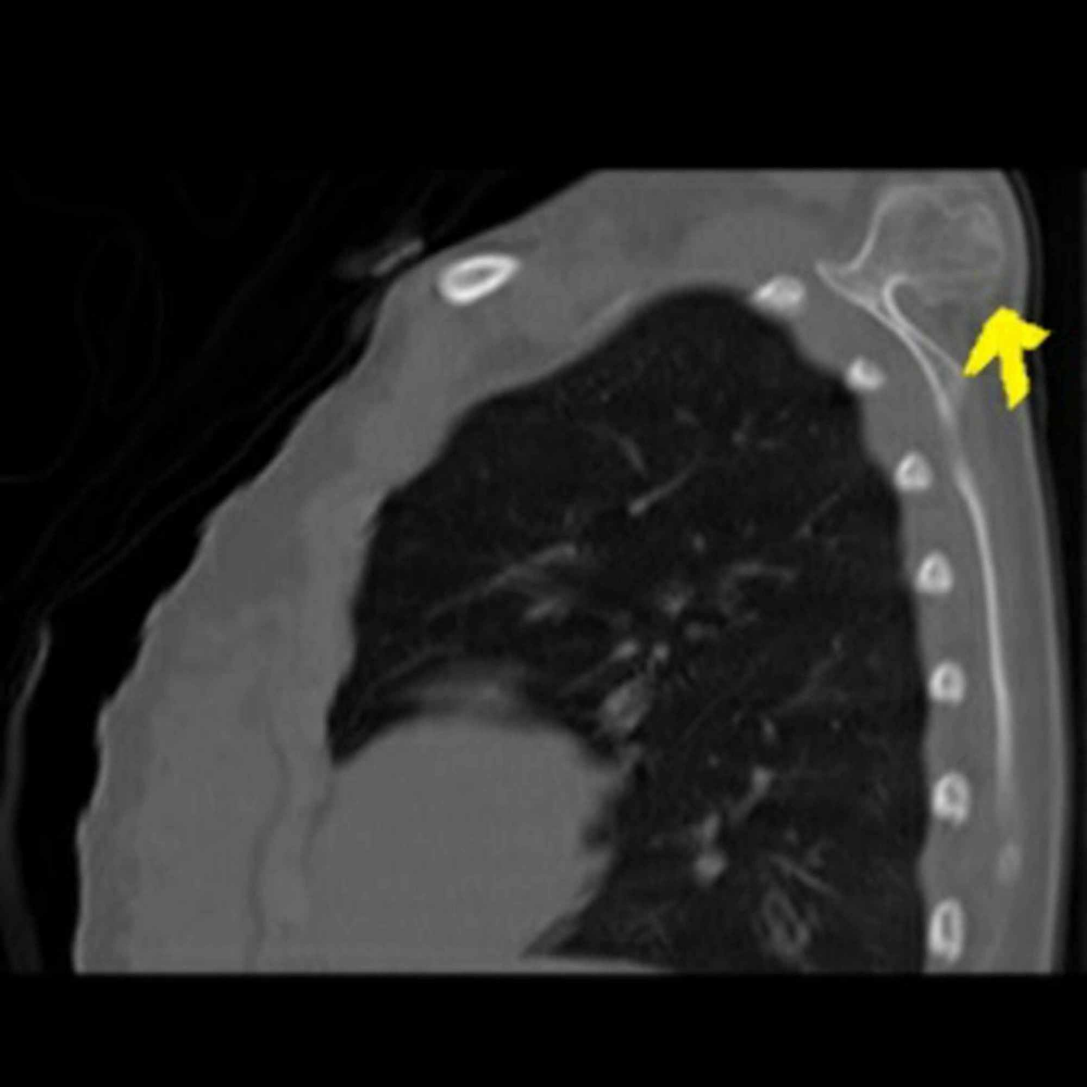
CT detected a pedunculated mass on dorsal aspect of the scapula.

A 15-year-old female patient was admitted to the Orthopedics and Traumatology Department with the complaint of a mass on the left upper back for five years. The patient complained of the inability to sleep in a supine position, pain with shoulder motion, and cosmetic discomfort for two years. She reported that there had been no growth in the size of the mass in the last three years. The patient did not have a history of trauma, family history, a history of osteochondroma in another region, weight loss, systemic signs, or any other known disease. On examination, a hard, oval solid mass was palpable with a size of about 6×4 cm on the superior-medial dorsum of the scapula (Figure 1). The mass was fixed, and the margins of the mass were prominent and they moved with the scapula. Abduction and extension of the shoulder were restricted and painful. There was no neurologic deficit in the upper limb. X-rays of the left shoulder revealed a bony mass arising from the dorsal aspect of the left scapula (Figure 2). A pedunculated mass was detected with a computed tomography (CT) scan on the dorsal aspect of the scapula (Figure 3). The patient was informed, and surgery for excisional biopsy was planned.

**Figure 4:**
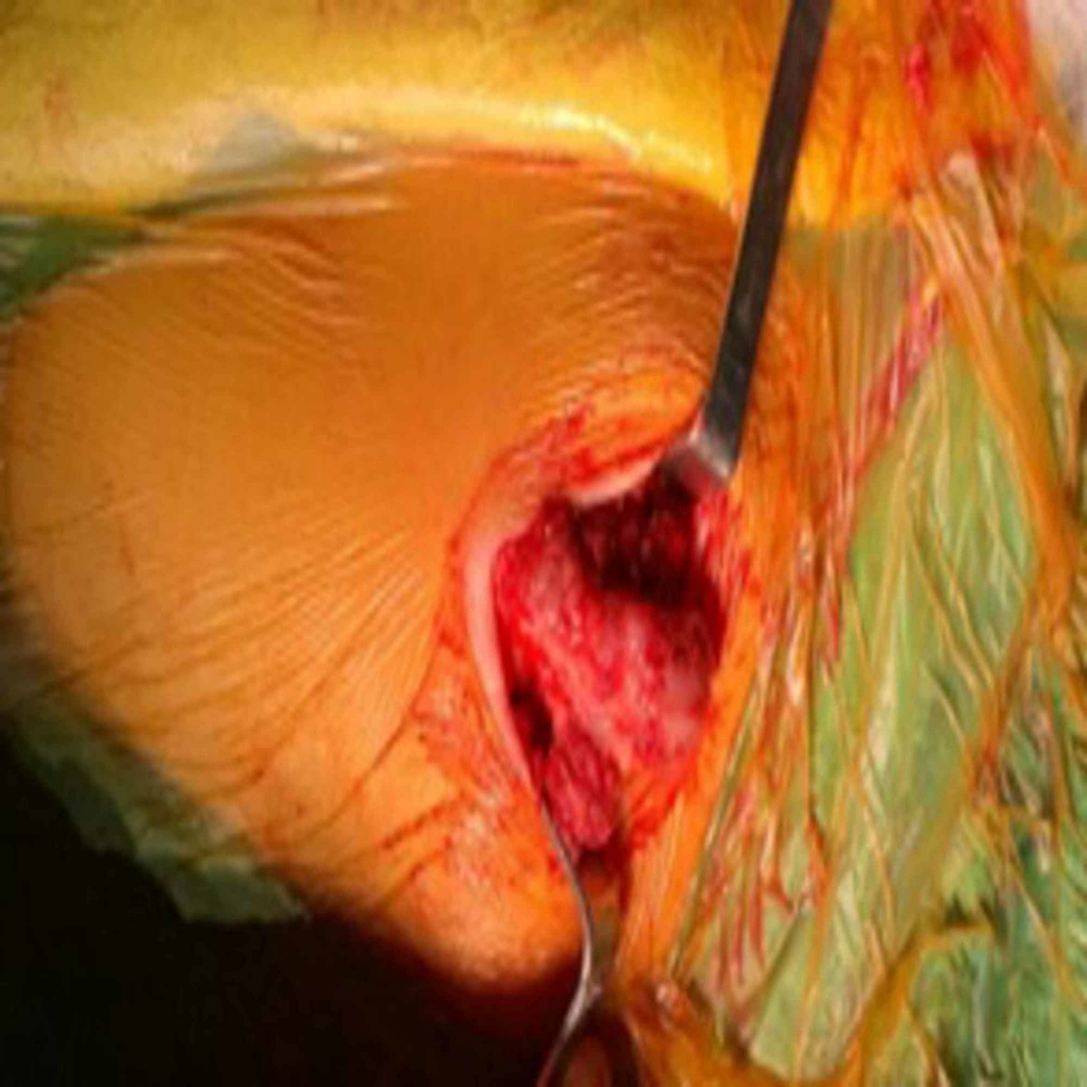
Intraoperative appearance of the scapular osteochondroma.

**Figure 5:**
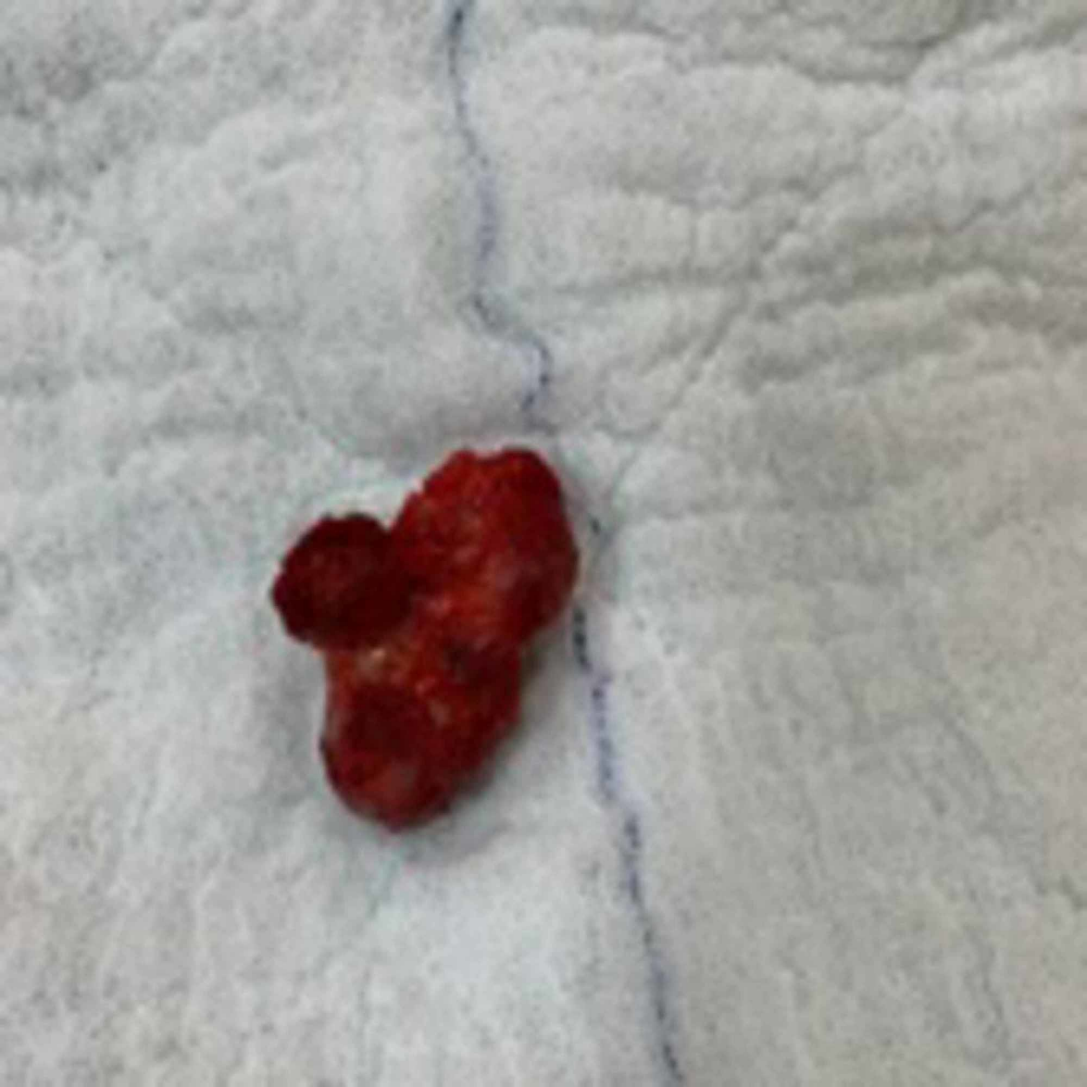
The specimen was sent for histopathologic examination.

Under general anesthesia, the patient was placed in the prone position. After cleaning the operation field with iodine, a sterile drape was applied. An incision was made over the mass. The soft tissues were dissected carefully and the borders of the mass were exposed (Figure 4). The peduncle was identified, and the mass was excised using an osteotome. The specimen was sent for histopathologic examination (Figure 5). The histopathologic investigation confirmed the diagnosis of non-malignant transformation osteochondroma. The cartilaginous cap was 2 mm. Non-steroidal anti-inflammatory drugs were ordered for pain relief. On the postoperative third day, the patient was discharged without any complications. After the operation, an arm sling was recommended for two weeks. Shoulder exercises were initiated after suture removal. At one-year follow-up, the patient’s complaints had completely regressed and no recurrence was observed. The patient has given informed consent for the case report to be published.

## Discussion

Osteochondromas are benign tumors that commonly affect the proximal humerus, pelvis, and knee but are rarely seen on flat bones [1]. They are usually asymptomatic. While patients with osteochondroma of the dorsal scapula are admitted for cosmetic reasons and due to the inability to sleep in a supine position, the main reason for admission in cases of ventral scapular osteochondromas is often mechanical symptoms (snapping, pseudo-winging) [3,5,6,8]. In our case, the patient had pain and difficulty with sleeping in a supine position. X-rays are usually sufficient for diagnosis. CT scans are helpful to diagnose volar scapular osteochondromas.

There have been some cases of solitary osteochondroma of the scapula reported in the literature. In most cases, the affected side was the volar aspect of the scapula [2,3,5-8]. Symptomatic solitary osteochondroma is rare on the dorsal aspect of the scapula.

Nathan et al. [11] performed a retrospective review of all osteochondroma excisions at their institution from 1994 to 2007. They presented five patients with lesions arising from the ventral surface of the scapula, two patients arising from the dorsal surface, and one patient in the inferior acromion. No signs of recurrence were reported in seven patients at a mean 4.17 years postresection period (88%). A single patient with a recurrence underwent two additional surgical procedures. 

Yadkikar and Yadkikar [12] presented a case of a dorsal scapular osteochondroma, where the patient complained of having difficulty sleeping in the supine position. Similarly, in a study by Salgia et al. [13], a patient was treated and was mainly concerned about cosmetic appearance.

In another case presentation, Jadhav et al. [10] presented two cases of osteochondroma. The first case was located at the dorsal aspect of the right scapula with a size of 3-4 cm. The patient was a 12-year-old male with a complaint of difficulty in sleeping in the supine position. X-ray of the scapula showed sessile swelling. The swelling was excised and at one-year follow-up, no recurrence was observed and the patient also improved symptomatically. Our case was a 15-year-old female who had the same complaints with a mass that had a size of 6×4 cm. The second case was located at the base of the first metatarsal.

Nekkanti et al. [9] presented two patients with osteochondroma on the dorsal surface of the scapula. The first patient was a 19-year-old male with complaints of discomfort while sleeping and moving his left shoulder for one year. Our case had similar complaints for two years. In the second case presentation, they reported a five-year-old male with swelling over the dorsal aspect of the left shoulder from birth. On physical examination, they detected a hard, oval bony swelling with a size of 1.5×1 cm. CT scan of the shoulder revealed a pedunculated osteochondroma. As in our study, both these cases were pedunculated osteochondroma.

## Conclusions

Osteochondromas may cause different symptoms depending on their localization. While osteochondroma is not common in the scapula, it should be kept in mind that the most common benign tumor of the scapula is osteochondroma. Although osteochondroma is more common in the ventral region and causes clinical snapping scapula, the dorsal involvement of the osteochondroma may also cause clinical symptoms depending on the size of the mass.
